# Integrated aquatic and terrestrial food production enhances micronutrient and economic productivity for nutrition-sensitive food systems

**DOI:** 10.1038/s43016-023-00840-8

**Published:** 2023-09-04

**Authors:** Liz Ignowski, Ben Belton, Hazrat Ali, Shakuntala Haraksingh Thilsted

**Affiliations:** 1 WorldFish, Phnom Penh, Cambodia; 2 Michigan State University, East Lansing, MI USA; 3 International Food Policy Research Institute, Dhaka, Bangladesh; 4 WorldFish, Dhaka, Bangladesh; 5 Consultative Group on International Agricultural Research, Washington, DC USA

**Keywords:** Agriculture, Developing world

## Abstract

Integrated aquaculture–agriculture (IAA) is a form of crop diversification where aquatic and terrestrial foods are grown together on a single parcel of land. We compare economic and nutrient productivity per hectare for 12 distinct IAA combinations, identified from a representative survey of 721 farms in southern Bangladesh. Just under half of households integrate agriculture into their aquaculture production. Regression analyses show positive associations between the integration of terrestrial foods into aquatic farming systems and nutrient productivity, but that nutrient productivity is partly disconnected from economic productivity. However, we find that production of specific combinations of aquatic foods and vegetables can simultaneously improve nutrient productivity and economic productivity, thereby promoting nutrition-sensitive agriculture (NSA). The approach demonstrated here can be applied to the design of NSA programmes that are important for realizing nutrition-sensitive food systems.

## Abstract (web summary)

Data from integrated aquaculture–agriculture farms in Bangladesh indicate that the production of specific combinations of aquatic foods and vegetables can simultaneously improve nutrient and economic productivity and promote nutrition-sensitive agriculture.

## Main

The green revolution—a bundle of improved plant varieties, inorganic fertilizer and improved irrigation, introduced to much of Asia during the 1970s and 1980s—sought to address hunger by enhancing staple crop yields to increase the availability of grains and raise farm incomes^1^. It was largely successful in achieving these objectives. For instance, Bangladesh went from experiencing a severe famine in 1974 in which 1.5 million people died to becoming the third-largest rice producer in the world, able to grow sufficient rice to feed its population of 169 million^2^.

However, malnutrition remains persistent in many countries that experienced the green revolution, despite large increases in staple crop production per hectare of arable land and per capita over the past half century. Growing awareness of this disconnect^3^ has led to calls for nutrition-sensitive approaches to agriculture and food systems that emphasize increasing consumption of micronutrient-rich foods, as opposed to prioritizing meeting energy needs with staple grains^4^. Nutrition-sensitive agriculture (NSA) programmes are designed to address the underlying determinants of malnutrition within a population and incorporate specific nutrition goals. For instance, NSA aims to improve diets in ways that include biofortification, home garden food production, livestock transfer programmes, more efficient value chains for nutritious foods and irrigation programmes^5^. Such programmes are often aimed at rural smallholder households, which are the focus of this Article.

Food production and income are two of the main pathways linking agriculture to household nutrition^5,6^. Promoting production of diverse foods is one component of NSA, reflecting evidence that increasing farm production diversity may improve smallholder farm household diets and nutrition^7^. Research from Bangladesh supports this view. For example, randomized control trials provided evidence that trainings on agriculture improved production diversity, while trainings on agriculture and nutrition improved both production diversity and diet quality in terms of quantity as measured by calories and quality as measured by the Global Diet Quality Score^8^. Other research from Bangladesh has found that interventions that raised farm incomes contributed to more diverse and nutritious diets by facilitating the purchase of non-staple foods^9^.

Increasing the production of nutritious foods may create spillovers beyond the farm by increasing the availability and accessibility of these foods to non-producer households through markets. Increases in farm income associated with production of food for the market may also diversify dietary intakes by allowing households to purchase foods that they are unable to produce or by smoothing seasonal variations in food availability on farm^10^. Other pathways to instituting more nutrition-sensitive food systems include behaviour change communication and cross-sectoral policy integration^11^. However, evidence on the efficacy of crop diversification as an NSA strategy is mixed^12^, and there are still important knowledge gaps to be filled, including questions regarding the sustainability, scale-up and cost effectiveness of NSA.

Aquaculture, the farming of aquatic organisms, has boomed in Bangladesh since the 1990s, induced by demand from rapidly growing domestic and export markets^13,14^. Aquatic foods are typically nutritious and economically valuable relative to staple foods. Integrated aquaculture–agriculture (IAA), where aquatic and terrestrial foods are grown together on the same plot, has been promoted widely to enhance production diversity, land productivity and nutrient cycling on farm, including in Bangladesh, where a wide variety of forms of IAA are practiced^15–17^. Examples include growing rice, fish and crustaceans in the same plot, concurrently or in rotation; growing climbing vegetables on frames built over ponds; and planting fruit trees or coconuts on pond banks^18^. Recent research has found that household engagement in aquaculture and horticulture simultaneously is associated with higher diet quality than either alone^19^. However, to date, little attention has been paid to whether IAA practices enhance production of micronutrients by smallholder farmers^20^.

This research gap motivates our paper, which presents a methodology for measuring the economic productivity and nutrient productivity of farming systems, and identifying complementarities and trade-offs between these outcomes. We focus on the total production of nutrients per hectare of land and water, rather than on the nutrient intakes of farm households, because many of the most nutritious foods (for example, fish, vegetables) are produced predominantly for sale, and because farm households in Bangladesh purchase the majority of the foods that they consume^21^. Our analysis contributes to understanding of the production of nutrients from diverse farming systems but does not assess the allocation of nutrients between producers and consumers.

## Results

We analyse data from a representative survey of 721 farms in southern Bangladesh, spanning a wide range of IAA practices. We combine data on the production of 35 aquatic and 31 terrestrial foods harvested from these farms over the most recently completed cropping cycle (a period of approximately one year) with food composition data to estimate the productivity per hectare of energy, protein and five key micronutrients that are both critical for human health and commonly deficient^22–24^: calcium, iron, zinc, vitamin A and vitamin B12.

We express economic productivity as the annual value of food production (US$ ha^−1^), calculated as income received from sales of food produced, plus the imputed value of any self-produced food consumed, minus the variable costs of food production. Nutrient productivity is expressed in annual adult equivalents per hectare (AEs ha^−1^) for 12 combinations of IAA. AEs are equivalent to the number of adults whose requirements for a specific nutrient could be met from one hectare for a period of one year. We use ordinary least squares regressions to estimate correlations between production of aquatic and terrestrial foods and economic value and nutrient production. This information can be used to make regionally specific recommendations for promoting NSA strategies such as IAA, based on empirical evidence from existing farmer practices.

Foods with the highest potential to maximize economic value include crustaceans and carps, while foods most associated with production of multiple micronutrients of interest include unstocked fish (mainly indigenous fish species that enter ponds when water is exchanged with the surrounding environment), green leafy vegetables and nuts/oilseeds.

Table 1 presents the distribution of 721 farms in our sample by farming system, defined in terms of four combinations of aquatic foods and four combinations of terrestrial foods. Farming systems with 12 or fewer observations were excluded, leaving 700 farms in our main analysis. Among aquatic food combinations, production of only fish is most common (39%), followed by fish, prawn and shrimp (29%); fish and prawn (26%); and fish and shrimp (8%). Among the households in our sample, 96% produce some carp species, 83% produce unstocked fish species, 82% produce other stocked fish and 59% produce crustaceans.

**Table 1:** Sample distribution by farming system

Terrestrial foods	Aquatic foods				
Form of integration	Fish (F)	Fish and prawn (FP)	Fish, prawn, shrimp (FPS)	Fish and shrimp (FS)	Total
Non-integrated (none)	184	32	135	52	403
Rice	16	33	29	8	86
Vegetables and fruits	68	31	12	1	112
Rice, vegetables and fruits	16	69	35	0	120
Total	284	165	211	61	721

More than half of households (56%) do not integrate agriculture into their aquaculture production. Integration of aquatic foods with vegetables and fruits and rice is the most common form of IAA (17% of farms), followed by integration with only vegetables and fruits (16%) and integration with only rice (12%). Potential for integration of terrestrial foods is related to the type of aquaculture practiced. Prawn is produced in freshwater or low-salinity environments and are thus well suited to integration with terrestrial foods. Shrimp is produced in saline water that damages terrestrial food crops, making crop integration more difficult. However, there is overlap in the range of salinities in which both crustacean species can thrive, giving rise to a diverse mix of IAA^25^. Most households producing prawns integrate with agriculture (81%), whereas IAA is only moderately common for households that produce only fish (35%) and comparatively rare for those producing shrimp (15%).

Research on the effects of salinity on diets in southern Bangladesh is somewhat ambiguous, suggesting that the interplay of salinity, production diversity, agricultural commercialization, subsistence capacity and dietary quality are complex. Numerous studies have found that salinization associated with shrimp aquaculture has negatively impacted food security and food sovereignty by inhibiting cultivation of terrestrial crops including rice, vegetables and livestock^26–28^. However, a recent study in southern Bangladesh finds that shrimp-farming households in saline areas have notably higher dietary diversity than non-shrimp-farming households in freshwater areas because the former have higher average incomes per capita, allowing them to offset lower levels of subsistence production by purchasing food^29^. Other recent research indicates that production of vegetables in IAA in southern Bangladesh is positively associated with dietary quality and inversely related to salinity levels but that average per capita intakes of vegetables, fruit and milk are similar among farm households across the salinity gradient due to good levels of market access^17^.

The diversity of production in our sample varies by farming system and by the combinations of foods produced on each farm. Farms harvested an average of nine aquatic products each, out of a total 35 produced, while the 32% of households that produced vegetables and fruits harvested 3.5 types each on average, out of a total 30 produced. Supplementary Fig. 1 depicts the study zone, with the location of the different farming systems surveyed.

**Fig. 1:**
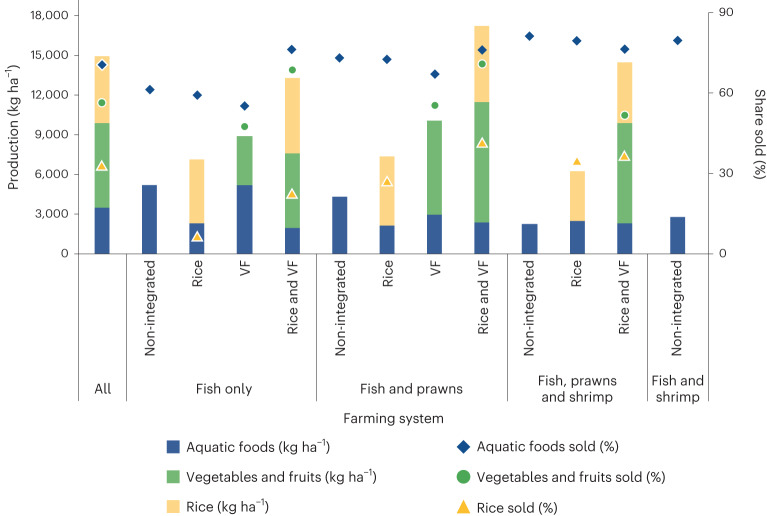
Average quantities of foods produced and shares sold for each food group in each farming system. Quantities of aquatic foods, rice and vegetables and fruits produced (kg ha^−1^) and shares sold (%) by farming system. VF, vegetables and fruits.

Figure 1 summarizes the average quantities of aquatic foods, vegetables and fruits and rice produced per hectare and the share of production sold for each food group in each farming system. The farming systems all combine income generation and subsistence, indicating two distinct agriculture–nutrition pathways. Households practicing non-integrated forms of aquaculture produce the lowest total quantities of food per hectare on average but some of the highest amounts of aquatic foods. Across all farming systems, the share of aquatic foods sold is highest (averaging 71%), followed by vegetables and fruits (57% sold) and lowest for rice (33% sold).

**Fig. 2:**
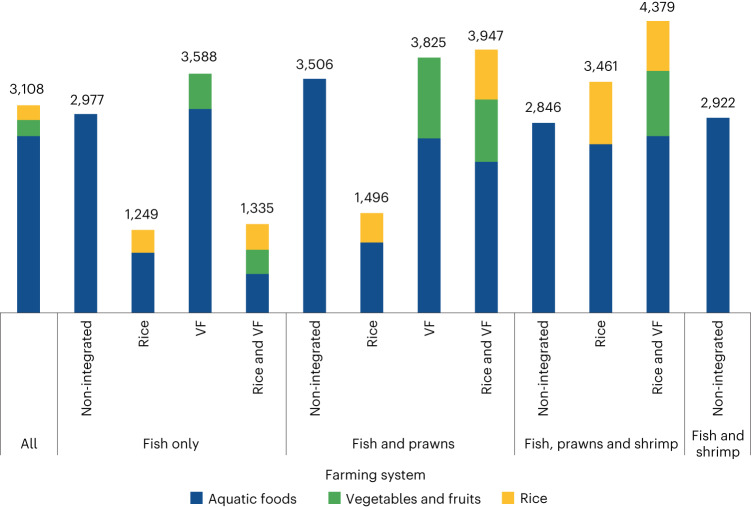
Economic productivity by farming system. Economic productivity (US$ ha^−1^) by farming system.

Figure 2 presents the economic productivity per hectare by farming system, disaggregated into aquatic foods, vegetables and fruits and rice. The most profitable farming systems produce fish, prawns and shrimp with rice, vegetables and fruits (US$4,379 ha^−1^) and fish and prawns with rice, vegetables and fruits (US$3,947 ha^−1^). The least economically productive farming systems produce fish integrated with rice (US$1,249 ha^−1^) and fish integrated with rice, vegetables and fruits (US$1,335 ha^−1^).

**Fig. 3:**
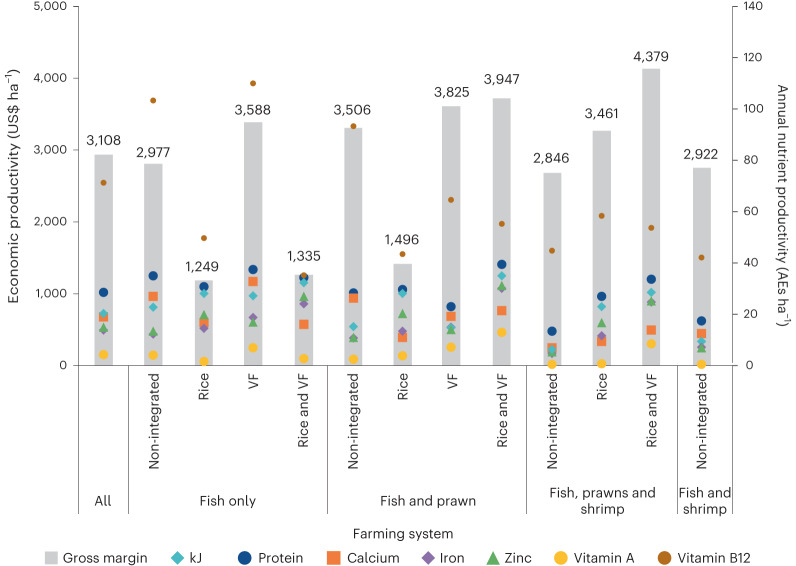
Economic productivity and estimates of nutrient productivity by farming system. Economic productivity (US$ ha^−1^) and nutrient productivity (AE ha^−1^ of kilojoules (kJ), protein and selected minerals and vitamins) by farming system.

Figure 3 extends this comparison by presenting economic productivity per hectare, overlaid with estimates of productivity of energy, protein, calcium, iron, zinc, vitamin A and vitamin B12, expressed as AEs ha^−1^ by farming system. The figure shows that the nutrient productivity of farming systems is partly disconnected from their economic productivity. IAA systems that combine fish and prawns with vegetables and fruits and rice—one of the most economically productive food combinations—also have the highest productivity of energy, protein, iron, zinc and vitamin A. However, whereas the economic productivity of farming systems that include shrimp but are not integrated with terrestrial foods is close to the sample average, these systems supply much lower than average quantities of almost all nutrients per hectare. These results point to positive associations between the integration of terrestrial foods into aquatic farming systems and nutrient productivity.

Supplementary Fig. 2 presents nutrient productivity results per farming system at higher resolution, indicating the share of nutrients derived from the three main food groups included in our analysis—aquatic foods, vegetables and fruits and rice. Rice supplies approximately three-quarters of energy in all farming systems in which it is produced. Rice is also an important source of protein, supplying approximately half of total protein in systems where it is cultivated. Aquatic foods are the major source of calcium across all farming systems, with a small amount of calcium originating from vegetables and fruits and minimal quantities from rice. In farming systems that integrate terrestrial foods, aquatic foods tend to provide lower shares of total iron than rice and/or vegetables and fruits. Similar results are found for zinc, with systems that produce the most iron also producing the most zinc. Rice accounts for the largest share of total zinc in all farming systems that produce rice. Vegetables and fruits are the main source of vitamin A for households that produce them, with aquatic foods contributing moderate levels of vitamin A in most farming systems. Iron is available from all three food groups studied. Vitamin B12 is only available from animal-source foods and therefore only originates from the aquatic foods in our sample. Farms producing fish integrated with vegetables and fruits or fish without terrestrial food integration produce the most vitamin B12. These systems produce the largest quantities of carp, some species of which have very high levels of vitamin B12.

## Regression analysis

To analyse these relationships with greater precision, we further sub-divide foods into four sub-categories of aquatic food and eight sub-categories of terrestrial food and regress economic productivity (US$ ha^−1^) and productivity of the seven nutrients of interest (AEs ha^−1^) against the quantities of each group of foods produced (descriptive statistics for the amount of each food produced can be found in Supplementary Table 1). Table 2 presents the regression results. These results do not imply causation but show higher resolution correlations between production of different foods and nutrient productivity, with the addition of various control variables. One important control variable included is farm size in hectares. Average farm size is small at 0.78 ha. We find no association between farm size and whether households integrate aquaculture with agriculture.

**Table 2:** Regression analysis: correlates of economic and nutrient productivity

*Production* (t ha^−1^)	Economic productivity (US$ ha^−1^)	kJ (AEs ha^−1^)	Protein (AEs ha^−1^)	Calcium (AEs ha^−1^)	Iron (AEs ha^−1^)	Zinc (AEs ha^−1^)	Vitamin A (AEs ha^−1^)	Vitamin B12 (AEs ha^−1^)
	(1)	(2)	(3)	(4)	(5)	(6)	(7)	(8)
***Carp***	**1,387.3**^c^	3.5^c^	**7.3**^c^	**6.5**^c^	2.6^c^	2.9^c^	0.2^a^	**25.6**^c^
	(60.8)	(0.1)	(0.0)	(0.3)	(0.1)	(0.1)	(0.1)	(1.2)
***Other stocked fish***	**438.1**^c^	**7.2**^c^	**7.1**^c^	2.4^c^	1.8^c^	2.0^c^	1.1^c^	**21.9**^c^
	(45.3)	(0.1)	(0.0)	(0.2)	(0.1)	(0.0)	(0.1)	(0.9)
***Unstocked fish***	−66.5	**4.6**^c^	**7.7**^c^	**12.5**^c^	**5.0**^c^	**4.2**^c^	**3.7**^c^	−1.3
	(369.4)	(0.8)	(0.3)	(1.7)	(0.7)	(0.4)	(0.7)	(7.4)
***Crustaceans***	**3,944.3**^c^	0.8^b^	5.0^c^	0.8	2.2^c^	2.4^c^	−0.3	**30.1**^c^
	(166.7)	(0.4)	(0.1)	(0.8)	(0.3)	(0.2)	(0.3)	(3.3)
***Rice***	47.7	**4.4**^c^	3.4^c^	0.4	2.0^c^	3.1^c^	0	0.4
	(80.1)	(0.2)	(0.1)	(0.4)	(0.1)	(0.1)	(0.1)	(1.6)
***Leafy vegetables***	64.6	−0.3	1.8^c^	3.3	**3.4**^c^	**3.3**^c^	**10.4**^c^	0.9
	(563.5)	(1.3)	(0.4)	(2.6)	(1.0)	(0.5)	(1.0)	(11.2)
***Vitamin A-rich vegetables***	336.1^c^	0	0.6^c^	1.3^c^	1.1^c^	0.2^b^	**8.9**^c^	0
	(106.8)	(0.2)	(0.1)	(0.5)	(0.2)	(0.1)	(0.2)	(2.1)
***Other vegetables***	142.7^c^	0.3^c^	0.6^c^	0.3^b^	1.3^c^	0.9^c^	0.3^c^	−0.1
	(26.1)	(0.1)	(0.0)	(0.1)	(0.0)	(0.0)	(0.0)	(0.5)
***Root crops***	−1,066.6	0.8	−1.2	3.6	−2.2	**4.0**^b^	−3.2	−0.8
	(2,087.4)	(4.8)	(1.6)	(9.6)	(3.8)	(2.0)	(3.7)	(41.7)
***Vitamin A-rich fruits***	−378.9	2.1^c^	−0.2	0	0.1	0.1	1.2^b^	−4.3
	(308.0)	(0.7)	(0.2)	(1.4)	(0.6)	(0.3)	(0.6)	(6.1)
***Other fruits***	132	1.0^c^	0.4^c^	−0.2	1.1^c^	0.4^c^	0.2	0
	(108.8)	(0.2)	(0.1)	(0.5)	(0.2)	(0.1)	(0.2)	(2.2)
***Nuts/oilseeds***	414.1^b^	3.8^c^	2.0^c^	**5.7**^c^	**6.7**^c^	1.6^c^	1.5^c^	3.4
	(161.7)	(0.4)	(0.1)	(0.7)	(0.3)	(0.2)	(0.3)	(3.2)
***Aquatic food sold (%)***	−240	−0.5	0	−6.1^b^	−2.0^a^	−0.3	0.9	1.7
	(587.7)	(1.3)	(0.4)	(2.7)	(1.1)	(0.6)	(1.1)	(11.7)
***Sold F and V (0/1)***	143.7	−0.3	0.1	−2.2	−0.9	0.8^b^	−0.8	−4
	(400.1)	(0.9)	(0.3)	(1.8)	(0.7)	(0.4)	(0.7)	(8.0)
***Sold rice (0/1)***	478.2	−0.2	0	−0.9	−0.7	−0.8^a^	−0.6	2.5
	(498.2)	(1.1)	(0.4)	(2.3)	(0.9)	(0.5)	(0.9)	(9.9)
***Constant***	−2,350.6^c^	3.6^a^	−1.1	4.2	2.5	−0.3	1.7	−48.1^c^
	(862.1)	(2.0)	(0.6)	(4.0)	(1.6)	(0.8)	(1.5)	(17.2)
***R squared***	0.79	0.92	0.99	0.68	0.87	0.95	0.82	0.72
***Observations***	**700**	**700**	**700**	**700**	**700**	**700**	**700**	**700**

Note: The dependent variable is the number of annual AEs of each nutrient produced per hectare. V and F, vegetables and fruits. All models are ordinary least squares regressions that control for household head age, household head education, if the household head is female, number of household members, the dependency ratio, a binary indicator of whether the household has off-farm income, travel time to nearest city, the hectares of agricultural land and ponds operated by the household and fixed effects at the upazila (subdistrict) level. The three coefficients with the largest statistically significant correlations are bolded and underlined within each specification.

^a^*p* < 0.10,

^b^*p* < 0.05,

^c^*p* < 0.01. Find the full table of results in Supplementary Table 2.

Model 1 in Table 2 demonstrates a positive and significant relationship between the productivity of three out of four aquatic food groups and economic productivity, with yields of crustaceans having the largest positive correlation with economic productivity, followed by yields of carp species. Yields of other stocked fish species, nuts/oilseeds and vitamin A-rich vegetables are also positively and significantly associated with economic productivity.

We find multiple positive and significant correlations between the productivity of different foods and productivity of nutrients. For each nutrient of interest (Models 2–8), the three coefficients with the largest statistically significant correlations are bolded and underlined. Unstocked fish species are strongly associated with the productivity of multiple key micronutrients. Productivity of green leafy vegetables is highly significantly associated with productivity of iron, zinc and vitamin A, as is the production of vitamin A-rich vegetables and other vegetables, but with a smaller coefficient. Production of vitamin A-rich fruits and other fruits has smaller and/or insignificant correlations with most nutrients, as do root crops. The yield of nuts/oilseeds is highly positively correlated with calcium and iron productivity. As expected, rice is an important source of energy and plant protein.

Further disaggregation of results (Supplementary Table 5) shows that two unstocked small fish species, mola and *tengra*, are particularly important sources of micronutrients. Consumption of small fish species such as these has been shown to improve intakes of calcium, iron and vitamin A in Bangladesh^30^. This result underlines the importance of the region’s aquatic biodiversity in supporting human nutrition^31,32^, with the Sundarbans (the world’s largest contiguous area of mangrove), located adjacent to the southwestern portion of the study area acting as a nursery ground for many of the unstocked aquatic species harvested.

Aquatic foods are far more economically valuable than terrestrial foods on average. Disaggregation of crustaceans (Supplementary Table 6) shows that three of five species included in this analysis are correlated with greater economic productivity. Unstocked fish are not economically productive in aggregate, but once this group is decomposed (Supplementary Table 5), we find that *tengra* and pool barb production correlate significantly with economic productivity. Further, many carp species are economically valuable, but *catla* is by far the most economically productive in our study, as seen in Supplementary Table 3.

Among vegetables and fruits, okra, gourds and long beans are all statistically significantly correlated with the productivity of protein, iron and zinc (Supplementary Table 7). Pumpkins (vitamin A-rich vegetables), *shak* (leafy vegetables), mangoes and betelnuts are important sources of vitamin A (Table 2 and Supplementary Tables 8 and 10). Coconuts are also positively statistically correlated with the productivity of energy and all nutrients in our study, except vitamin B12 (Supplementary Table 10).

These regressions demonstrate that the species and combinations of aquatic and terrestrial foods produced matter for economic and nutrient productivity and that the mix of aquatic foods and vegetables included in integrated farming systems could be key to optimizing economic productivity and nutritional adequacy. We find that aquatic foods are more nutritious per kilogram for certain nutrients but that their integration with terrestrial foods improves the overall availability of the nutrients included in this analysis. These results only relate to the production of foods, not the effects of their sale or consumption.

## Discussion

This paper contributes to a growing body of research on nutrition-sensitive food systems and NSA. Most literature on NSA to date has been conceptual^5,6,33^ or has evaluated the impact of planned nutrition-sensitive interventions on demand-side outcomes (for example, changes in household diet diversity or food consumption scores^7,34^). This paper’s key contribution is a supply-side methodology for estimating the nutrient productivity of farming systems.

Agricultural productivity is conventionally measured in terms of biomass or income per area of land. The present study introduces a nutrition-sensitive metric for agricultural productivity, expressed as production of kilojoules (kJ), protein and micronutrients, relative to human nutritional requirements (AEs ha^−1^). This approach allows us explore the relationship between economic and nutrient productivity across a range of existing IAA systems, identified inductively from a representative survey of 721 farms in southwest Bangladesh. The results provide an intuitive measure of nutrient sensitivity that may be easily understood by researchers and policymakers and mobilized by development practitioners and food producers.

We find strong empirical evidence that production diversity associated with integration of aquatic and terrestrial foods in IAA systems can be beneficial for both economic and nutrient productivity. This finding has important implications for the design of NSA programs to enhance the contributions that aquaculture makes to nutrition security in Bangladesh and other countries where IAA is commonly practiced and for the realization of nutrition-sensitive food systems.

The results can also be used to identify and promote culturally and agroecologically suitable combinations of foods that optimize nutritional and economic outcomes. For example, we find that common crops such as bitter gourds, bottle gourds and long beans are associated with high levels of nutrient productivity in addition to better-known vitamin A-rich crops such as green leafy vegetables.

However, increasing production diversity is not necessarily the most effective path to improving diet diversity^35,36^. Income is another pathway to nutrition, and households that earn money from economically productive but less nutritious foods such as crustaceans may use it to purchase nutritious foods instead of producing them^29^. This is a crucial point as shrimp (which are economically valuable) are produced in saline ponds, making integration with terrestrial crops challenging. It may be more beneficial for these households to seek to increase yields of shrimp and diversify production of aquatic crops to maximize income and aquatic source nutrients.

The approach presented in this paper can also be used to identify possible improvements to farming practices such as facilitating the entry of nutritionally and economically productive unstocked fish species into ponds or identifying suitable candidate fish species for domestication via investments in fish breeding research^37^. Future research using the methods developed here can also seek to identify and promote recommendations for specific crop combinations that maximize economic and nutrient output for a given level of salinity.

## Methods

This research complies with all relevant ethical regulations; the Michigan State University Institutional Review Board determined this study (STUDY00003689) to be exempt under 45 CFR 46.104(d) 2(ii). Survey data were collected in December 2020 and January 2021 using KoBoToolbox for the second round of a panel survey first conducted in 2013. In 2013, in each of seven selected districts, all sub-districts (*upazila*) with non-negligible aquaculture production were included in the initial sample frame, then selected randomly by proportional probability sampling. In each selected upazila, all *mouza* (the smallest administrative unit reported in the Bangladesh agricultural census), underwent a second stage of trimming to eliminate those with fewer than 20 aquaculture farms, as reported in the national agricultural census of 2008 (the most recently available agricultural census for Bangladesh). Two to three *mouza* were then selected randomly from each *upazila*. Before the survey, a census of fish farmers was conducted in all selected *mouza*, among which 20 farms were selected randomly for interview.

In 2020, we conducted a new farm census in each *mouza* included in the 2013 survey. All farms included in the previous survey round that could be contacted and gave their consent to be interviewed were resurveyed. The rate of attrition between the two survey rounds was approximately 20%. All missing farms were replaced at random with others selected from the updated census list. During the 2020 survey, detailed production data were collected from a single ‘sample parcel’ that had been used for aquaculture within the past 12 months, regardless of whether it was integrated with terrestrial foods. Where households operated more than one plot of aquaculture land, the sample parcel was selected at random from among these. The sample thus represents the entire population of aquaculture farms in the seven selected districts. All descriptive statistics and regression analyses were computed in StataSE Version 17.

We calculate the average production value per hectare for each farming system in US dollars (US$) by multiplying the reported quantity of each food produced (standardized per ha) by the unit price, as reported by households that sold aquatic foods. For terrestrial foods, unit prices were calculated by dividing the value of sales of each individual food by the amount sold, as reported by each farmer. Economic productivity is calculated by subtracting reported production costs per hectare from the value of production per hectare, including the imputed value of self-produced food consumed by the household. Production costs include stocking, inputs, harvesting and labour (both household and hired).

To estimate the nutritional value of production from each farm, we used the *Food Composition Table for Bangladesh*^38^ to calculate the nutritional value per kilogram of vegetables and fruits produced. The nutritional value of aquatic foods was calculated using data from Bogard et al.^39^. We estimate productivity of energy (measured in kilojoules), protein and the micronutrients calcium, iron, zinc, vitamin A and vitamin B12. These micronutrients are important for human growth, development and health and tend to have high levels of deficiencies in Bangladesh^40^.

To aid interpretation and comparability of the results, we express nutrient productivity as the number of adult equivalents whose complete annual dietary requirements could be met from the food produced on one hectare of land in one year (AEs ha^−1^). We estimate nutrient adequacy using recommended dietary allowances (RDAs), defined as the average daily dietary nutrient intake level that is sufficient to meet the nutrient requirements of nearly all (97–98%) healthy individuals for a given life stage and gender group^41^. We use RDA values for adults to estimate adult equivalent (AE) nutrient requirements. RDAs used in this analysis are 9,200 kJ, 55 g protein, 1,000 mg calcium, 13 mg iron, 10 mg zinc, 900 μg retinol activity equivalents vitamin A and 2.5 mg vitamin B12^41^. As energy requirements (kcal) cannot be calculated by the RDA method^42^, our value is approximated for a moderately active adult using details from several sources^41–43^.

To calculate the amount of nutrients produced, we multiply the weight of the food produced by its edible portion and nutrient concentration. To standardize these values, we divide the amount of each nutrient produced per ha by the amount that would equal one daily AE and multiply by 365. This calculates the number of annual AEs of each nutrient that the household produced per ha. For example, the average household in our sample produced enough energy per hectare for 22 adults to meet their energy requirements for a year.

### Reporting summary

Further information on research design is available in the Nature Portfolio Reporting Summary linked to this article.

### Supplementary information

Supplementary Information Supplementary Figs. 1 and 2 and Tables 1–10.

Reporting Summary

## Data Availability

The two nutrient datasets of the *Bangladesh Food Composition Table* and nutrient composition of fish species are publicly available at https://www.fao.org/fileadmin/templates/food_composition/documents/FCT_10_2_14_final_version.pdf and 10.1371/journal.pone.0175098. Survey data collected by WorldFish and used in this analysis will be publicly available via 10.7910/DVN/5HLK4G as of 26 March 2024 and until then it will be shared upon reasonable request to the corresponding author.

## Code availability

The statistical code will be publicly available at 10.5281/zenodo.8210239 as of 26 March 2024 or from the corresponding author on reasonable request.
