# Supplementary material for: Integrated aquatic and terrestrial food production enhances micronutrient and economic productivity for nutrition-sensitive food systems
Source: Nat Food. 2023 Sep 4;4(10):866–73. doi: 10.1038/s43016-023-00840-8 (PMC10589083; doi:10.1038/s43016-023-00840-8)
Supplement: Supplementary file 1 — Supplementary Figs. 1 and 2 and Tables 1–10. [file 43016_2023_840_MOESM1_ESM.pdf]

nature food

Article

https://doi.org/10.1038/s43016-023-00840-8

Integrated aquatic and terrestrial food production enhances micronutrient and economic productivity for nutrition-sensitive food systems

In the format provided by the authors and unedited

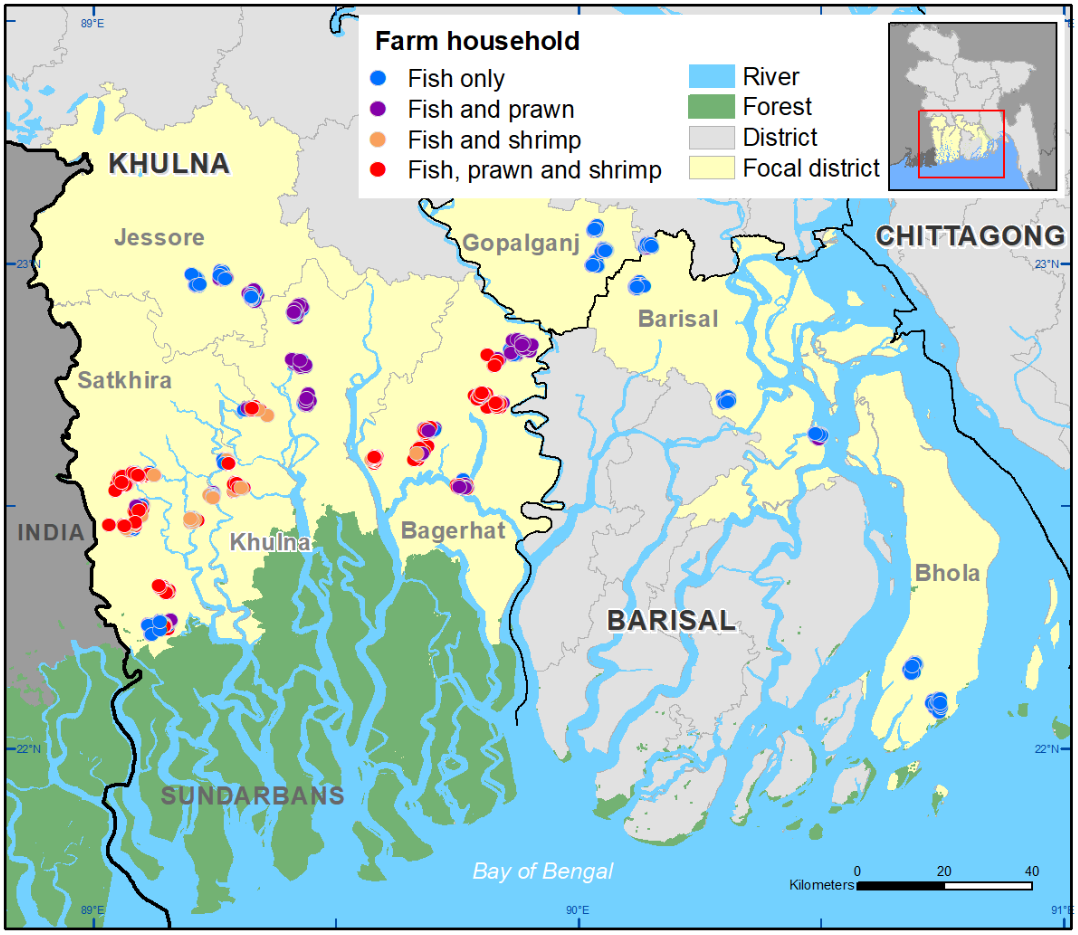

Note: Sampled farms were often near one another which leads to overlapping of points in Figure 1.
Prepared by Shwu Jiau Teoh, WorldFish using survey data

Figure 1: Map of study area.

Figure 2: Annual AEs for nutrients by food source and farming system per Ha.

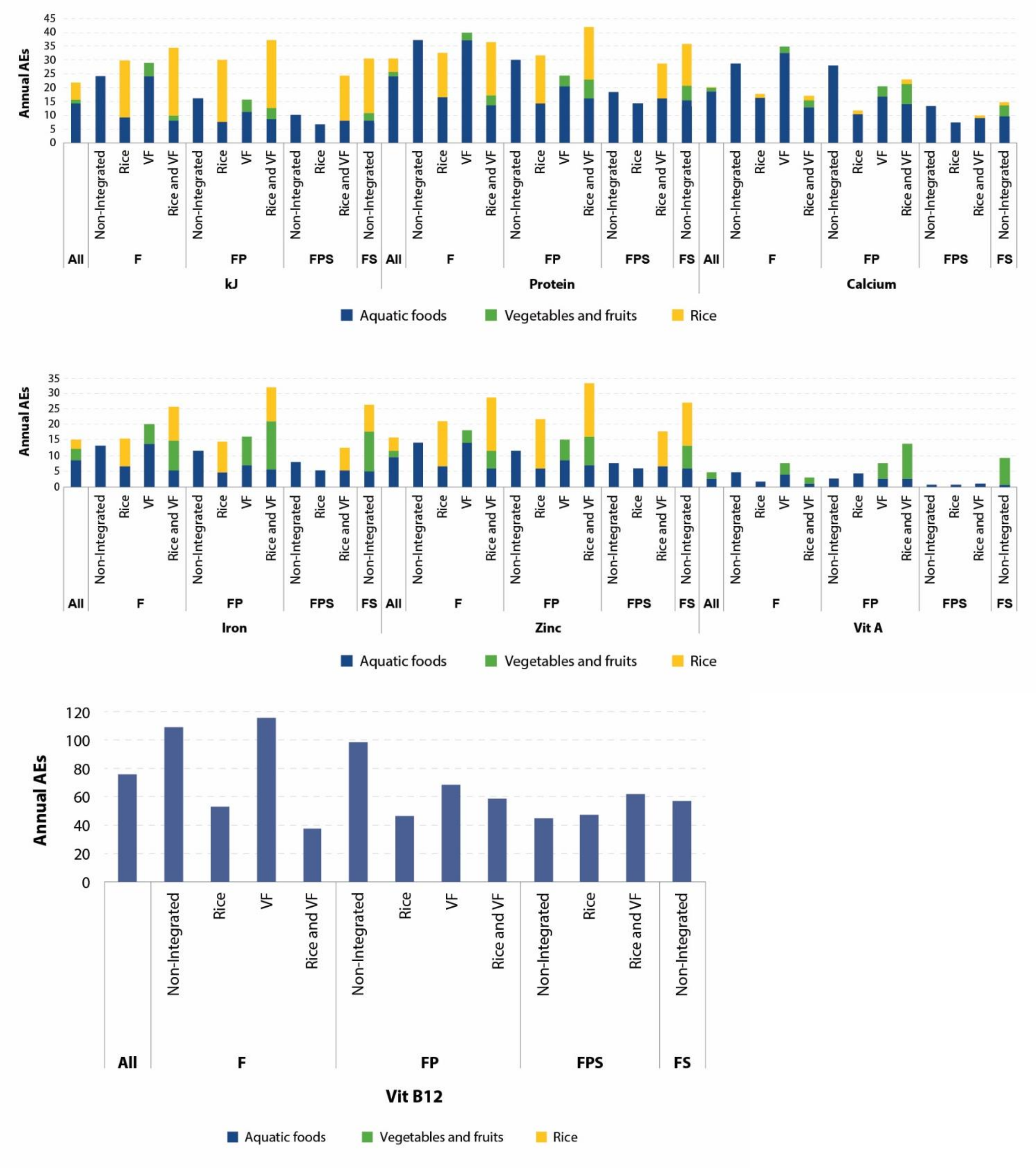

Scientific name	Common name	Obs	Mean production (kg/ha)	Price (USD/ kg)	Scientific name	Local name	Obs	Mean production (kg/ha)	Price (USD/ kg)
**Carp**					**Leafy vegetables**				
Labeo rohita	Rohu	643	704.2	1.60	Spinacia oleracea	Shak (any)	34	670.5	0.17
Catla	Katla	497	398.8	1.75	**Vitamin A-rich vegetables**				
Cirrhinus cirrhosus	Mrigal	400	436.4	1.36	Cucurbita moschata	Pumpkin	38	2,433.9	0.29
Labeo calbasu	Kalibaus	56	263.8	2.07	**Other vegetables**				
Hypophthalmichthys molitrix	Silver carp	300	566.6	1.10	Capsicum Chinense	Chili	35	191.3	1.40
Ctenopharyngodon idella	Grass carp	402	350.1	1.42	Solanum melongena	Egg plant	47	1,055.4	0.29
Cyprinus carpio	Common carp	431	373.5	1.35	Abelmoschus esculentus	Okra	26	449.1	0.27
Barbodes gonionotus	Silver barb	309	373.1	1.15	Luffa acutangula	Ridge gourd	20	1,718.0	0.26
Mylopharyngodon piceus	Black carp	29	92.3	1.70	Momordica charantia	Bitter gourd	54	2,749.5	0.38
**Other stocked fish**					Trichosanthes cucumerina	Snake gourd	8	1,963.3	0.26
Oreochromis niloticus	Tilapia	498	751.1	0.90	Lagenaria siceraria	bottle gourd	77	2,501.6	0.17
Anabas cobojius	Climbing perch	183	162.7	2.78	Solanum lycopersicum	Tomato	77	3,248.4	0.38
Pangasius	Pangasius	42	3,490.4	1.20	Cucumis sativus	Cucumber	66	3,208.8	0.28
Clarias batrachus	Walking catfish	12	3,333.1	3.45	Raphanus sativus	Radish	9	353.8	0.18
Heteropneustes fossilis	Stinging catfish	54	351.8	4.84	Brassica oleracea var capitata	Cabbage	12	777.8	0.16
**Unstocked fish**					Brassica oleracea var botrytis	Cauliflower	13	323.9	0.27
Chitala	Clown knifefish	5	147.9	4.76	Armoracia rusticana	Moringa	2	392.8	0.49
Channa striatas, Channa punctata	Snakehead	353	79.1	3.48		Others	7	773.2	0.005
Mystus tengara	Tengra	223	51.5	3.21	Vigna unguiculata	Cowpea	1	882.5	0.99
Lates calcarifer	Barramundi	125	134.5	4.77	Vigna unguiculata	Long bean	34	1,367.3	0.34
Labeo bata	Bata	39	310.0	1.32	**Roots**				
Amblypharyngodon mola	Mola	144	83.4	1.84	Solanum tuberosum	Potato	7	503.8	0.30
Puntius sophore	Pool barb	339	99.0	0.87	**Vitamin A-rich fruit**				
Mugil cephalus	Grey mullet	174	139.3	2.54	Carica papaya	Papaya	21	1,399.5	0.23
Boga labeo	Bhangan	39	125.6	4.15	Mangifera indica	Mango	26	1,158.9	0.58
Acanthopagrus datnia	Yellowfin seabream	14	60.4	3.41	**Other fruit**				
Scatophagus quadranus	Spotted scat	14	91.2	3.13	Musa acuminata	Banana	44	2,887.7	0.34
Rhinomugil corsula	Corsula	37	92.3	3.18	Artocarpus heterophyllus	Jackfruit	7	3,893.4	0.23
	Others	39	95.9	2.63	Psidium guajava	Guava	6	189.1	0.71
**Crustaceans**					Citrullus vulgaris Schrad	Watermelon	1	98.8	0.40
Penaeus monodon	Black tiger shrimp	237	415.1	6.38	Spondias pinnata	Hog plum	3	5,283.3	0.66
Macrobrachium rosenbergii	Giant freshwater prawn	361	235.0	8.11	**Nuts and Oilseeds**				
Fenneropenaeus indicus	Indian white prawn	9	40.5	4.70	Areca catechu	Betel nut	15	1,103.3	2.80
Metapenaeus Monoceros	Spotted shrimp	124	196.3	4.70	Cocos nucifera	Coconut	62	1,363.2	0.33
Scylla serrata	Mud crab	29	180.2	6.07	Brassica juncea	Mustard (seed)	2	2,121.5	1.63
**Note: Butter catfish, aire, mud eel, sweet potato, and chui were removed from table as no household produced these items.**					Oryza sativa	**Rice**	197	5,701.4	0.28

Table 1. Average quantity (Kg/Ha) and farmgate value (USD/Kg) of all aquatic and terrestrial foods produced by surveyed farms.

Production ***(t/ha)***	*Gross margin* ***(USD*/ha)**	kJ **(AEs/ha)**	*Protein* ***(AEs/ ha)***	*Calcium* ***(AEs/ ha)***	*Iron* ***(AEs/ ha)***	*Zinc* ***(AEs/ha)***	*Vitamin A* ***(AEs/ha)***	*Vitamin B12* ***(AEs/ha)***
**Carp**	1387.3***	3.5***	7.3***	6.5***	2.6***	2.9***	0.2*	25.6***
	(60.8)	(0.1)	(0.0)	(0.3)	(0.1)	(0.1)	(0.1)	(1.2)
**Other stocked fish**	438.1***	7.2***	7.1***	2.4***	1.8***	2.0***	1.1***	21.9***
	(45.3)	(0.1)	(0.0)	(0.2)	(0.1)	(0.0)	(0.1)	(0.9)
**Unstocked fish**	-66.5	4.6***	7.7***	12.5***	5.0***	4.2***	3.7***	-1.3
	(369.4)	(0.8)	(0.3)	(1.7)	(0.7)	(0.4)	(0.7)	(7.4)
**Crustaceans**	3944.3***	0.8**	5.0***	0.8	2.2***	2.4***	-0.3	30.1***
	(166.7)	(0.4)	(0.1)	(0.8)	(0.3)	(0.2)	(0.3)	(3.3)
**Rice**	47.7	4.4***	3.4***	0.4	2.0***	3.1***	0	0.4
	(80.1)	(0.2)	(0.1)	(0.4)	(0.1)	(0.1)	(0.1)	(1.6)
**Leafy vegetables**	64.6	-0.3	1.8***	3.3	3.4***	3.3***	10.4***	0.9
	(563.5)	(1.3)	(0.4)	(2.6)	(1.0)	(0.5)	(1.0)	(11.2)
**Vit. A-rich vegetables**	336.1***	0	0.6***	1.3***	1.1***	0.2**	8.9***	0
	(106.8)	(0.2)	(0.1)	(0.5)	(0.2)	(0.1)	(0.2)	(2.1)
**Other vegetables**	142.7***	0.3***	0.6***	0.3**	1.3***	0.9***	0.3***	-0.1
	(26.1)	(0.1)	(0.0)	(0.1)	(0.0)	(0.0)	(0.0)	(0.5)
**Root crops**	-1066.6	0.8	-1.2	3.6	-2.2	4.0**	-3.2	-0.8
	(2087.4)	(4.8)	(1.6)	(9.6)	(3.8)	(2.0)	(3.7)	(41.7)
**Vit. A-rich fruits**	-378.9	2.1***	-0.2	0	0.1	0.1	1.2**	-4.3
	(308.0)	(0.7)	(0.2)	(1.4)	(0.6)	(0.3)	(0.6)	(6.1)
**Other fruits**	132	1.0***	0.4***	-0.2	1.1***	0.4***	0.2	0
	(108.8)	(0.2)	(0.1)	(0.5)	(0.2)	(0.1)	(0.2)	(2.2)
**Nuts/oilseeds**	414.1**	3.8***	2.0***	5.7***	6.7***	1.6***	1.5***	3.4
	(161.7)	(0.4)	(0.1)	(0.7)	(0.3)	(0.2)	(0.3)	(3.2)
**HH head education**	69.8**	-0.1*	0	0	0	0	-0.1	1
	(33.3)	(0.1)	(0.0)	(0.2)	(0.1)	(0.0)	(0.1)	(0.7)
**HH size**	-58.3	-0.1	0.1**	0.2	0.2	0.2***	0.2	4.0**
	(80.4)	(0.2)	(0.1)	(0.4)	(0.1)	(0.1)	(0.1)	(1.6)
**HH head age**	5.9	0	0	0	-0.0*	0	-0.0*	0.2
	(10.2)	(0.0)	(0.0)	(0.0)	(0.0)	(0.0)	(0.0)	(0.2)
**HH has off farm income (0/1)**	324.7	-0.2	-0.3	1	-0.2	-0.1	-0.3	-0.6
	(260.2)	(0.6)	(0.2)	(1.2)	(0.5)	(0.2)	(0.5)	(5.2)
**HH head female**	716	-0.6	-0.2	-1.8	-1.1	-0.1	-0.1	24.7
	(840.4)	(1.9)	(0.6)	(3.9)	(1.5)	(0.8)	(1.5)	(16.8)
**Travel time to nearest city (min)**	-19.9*	-0.0*	0	-0.1	0	0	0	0
	(11.5)	(0.0)	(0.0)	(0.1)	(0.0)	(0.0)	(0.0)	(0.2)
**Share of fish sold**	-240	-0.5	0	-6.1**	-2.0*	-0.3	0.9	1.7
	(587.7)	(1.3)	(0.4)	(2.7)	(1.1)	(0.6)	(1.1)	(11.7)
**Sold F and V (0/1)**	143.7	-0.3	0.1	-2.2	-0.9	0.8**	-0.8	-4
	(400.1)	(0.9)	(0.3)	(1.8)	(0.7)	(0.4)	(0.7)	(8.0)
**Sold rice (0/1)**	478.2	-0.2	0	-0.9	-0.7	-0.8*	-0.6	2.5
	(498.2)	(1.1)	(0.4)	(2.3)	(0.9)	(0.5)	(0.9)	(9.9)
**Dependency ratio**	1037.5*	-1.6	0.3	4.8*	2.4**	0	1.3	14.1
	(621.1)	(1.4)	(0.5)	(2.8)	(1.1)	(0.6)	(1.1)	(12.4)
**Ag land (Ha)**	119.3	0.1	-0.4	2.7	0.4	0	0.9	5.8
	(432.0)	(1.0)	(0.3)	(2.0)	(0.8)	(0.4)	(0.8)	(8.6)
**Ponds (Ha)**	600.2***	0.1	-0.2*	0	-0.2	-0.2	-0.1	0.7
	(176.3)	(0.4)	(0.1)	(0.8)	(0.3)	(0.2)	(0.3)	(3.5)
**Constant**	-2350.6***	3.6*	-1.1	4.2	2.5	-0.3	1.7	-48.1***
	(862.1)	(2.0)	(0.6)	(4.0)	(1.6)	(0.8)	(1.5)	(17.2)
**R-squared within**	0.79	0.92	0.99	0.68	0.87	0.95	0.82	0.72
**Observations**	700	700	700	700	700	700	700	700

Note: The dependent variable is the number of annual AEs of each nutrient produced per hectare. All models OLS regressions that control for fixed effects at the upazila level. * p<0.10, ** p<0.05, *** p<.01.

Table 2: Regression analysis: correlates of economic and nutritional productivity, full results.

**Carp** / **Amount produced (t/ha)**	**Gross margin** (USD/ha)	**kJ** (Annual AEs/ha)	**Protein** (Annual AEs/ha)	**Calcium** (Annual AEs/ha)	**Iron** (Annual AEs/ha)	**Zinc** (Annual AEs/ha)	**Vitamin A** (Annual AEs/ha)	**Vitamin B12** (Annual AEs/ha)
	(1)	(2)	(3)	(4)	(5)	(6)	(7)	(8)
**Rohu**	371.4*	2.5***	8.5***	-0.3	1.8***	2.6***	-0.3	34.1***
	(197.6)	(0.5)	(0.2)	(0.8)	(0.4)	(0.2)	(0.4)	(4.0)
**Catla**	4894.1***	4.1***	5.6***	4.1***	1	2.1***	2.6***	39.4***
	(363.2)	(0.9)	(0.3)	(1.4)	(0.7)	(0.4)	(0.7)	(7.3)
**Mrigal**	1342.5***	4.0***	7.6***	22.5***	4.4***	3.4***	-0.4	46.6***
	(336.2)	(0.8)	(0.3)	(1.3)	(0.6)	(0.3)	(0.6)	(6.7)
**Kalibaus**	-220.8	5.7**	8.4***	-2.4	5.6***	0.2	3.6*	-3.6
	(1038.3)	(2.5)	(0.8)	(4.1)	(1.9)	(1.0)	(2.0)	(20.8)
**Silver carp**	248.3	4.2***	7.3***	17.6***	6.4***	3.0***	-0.9*	-6.3
	(289.4)	(0.7)	(0.2)	(1.1)	(0.5)	(0.3)	(0.6)	(5.8)
**Grass carp**	926.9**	4.3***	5.8***	1.8	0.6	1.7***	-0.7	4
	(379.7)	(0.9)	(0.3)	(1.5)	(0.7)	(0.4)	(0.7)	(7.6)
**Common carp**	568	4.3***	6.5***	1.2	1.9***	4.9***	-1.3*	-13.6*
	(374.9)	(0.9)	(0.3)	(1.5)	(0.7)	(0.4)	(0.7)	(7.5)
**Silver barb**	1136.9***	4.3***	7.6***	7.0***	2.3***	4.7***	1.6**	19.8***
	(370.5)	(0.9)	(0.3)	(1.5)	(0.7)	(0.4)	(0.7)	(7.4)
**Black carp**	-5603	6.3	7.0*	4.3	5.5	-0.5	11.9	42.3
	(4781.0)	(11.6)	(3.6)	(18.8)	(8.8)	(4.7)	(9.2)	(95.8)
**Observations**	**700**	**700**	**700**	**700**	**700**	**700**	**700**	**700**

Note: The dependent variable is the gross margin or number of annual AEs of each nutrient produced per hectare. All models are OLS regressions that control for production amounts of other food groups: other stocked fish, unstocked fish, crustaceans, rice, leafy vegetables, vitamin A vegetables, other vegetables, root vegetables, vitamin A fruit, other fruit, nuts and oilseeds, as well as household head education, number of household members, household head age, a binary indicator if the household has off farm income, if the household head is female, the travel time to nearest city, the dependency ratio, the hectares of agricultural land and ponds used by the households, and fixed effects at the upazila level. * p<0.10, ** p<0.05, *** p<.01.

Table 3: Regression analysis: correlates of economic and nutritional productivity, carp disaggregated.

**Other stocked fish**<br><br>**Amount produced (t/ha)**	**Gross margin** (USD/ha) (1)	**kJ** (Annual AEs/ha) (2)	**Protein** (Annual AEs/ha) (3)	**Calcium** (Annual AEs/ha) (4)	**Iron** (Annual AEs/ha) (5)	**Zinc** (Annual AEs/ha) (6)	**Vitamin A** (Annual AEs/ha) (7)	**Vitamin B12** (Annual AEs/ha) (8)
**Tilapia**	222.8**	4.1***	8.2***	2.0***	2.0***	2.7***	0.2*	8.5***
	(97.5)	(0.0)	(0.1)	(0.3)	(0.2)	(0.1)	(0.1)	(0.7)
**Climbing perch**	797.8***	8.0***	6.8***	1.6*	1.4***	1.3***	8.5***	24.1***
	(306.5)	(0.1)	(0.2)	(0.9)	(0.5)	(0.2)	(0.4)	(2.3)
**Pangasius**	475.3***	9.6***	6.6***	0.2	1.2***	1.5***	0.7***	13.7***
	(61.6)	(0.0)	(0.0)	(0.2)	(0.1)	(0.0)	(0.1)	(0.5)
**Walking catfish**	575.4***	3.5***	7.1***	11.4***	3.8***	2.8***	2.7***	61.4***
	(115.5)	(0.0)	(0.1)	(0.4)	(0.2)	(0.1)	(0.2)	(0.9)
**Stinging catfish**	767.6*	3.9***	8.1***	0.6	3.9***	2.8***	0.4	126.9***
	(429.0)	(0.1)	(0.2)	(1.3)	(0.7)	(0.3)	(0.6)	(3.2)
**Observations**	**700**	**700**	**700**	**700**	**700**	**700**	**700**	**700**

Note: The dependent variable is the number of annual AEs of each nutrient produced per hectare. All models are OLS regressions that control for production amounts of other food groups: carp, unstocked fish, crustaceans, rice, leafy vegetables, vitamin A vegetables, other vegetables, root vegetables, vitamin A fruit, other fruit, nuts and oilseeds, as well as household head education, number of household members, household head age, a binary indicator if the household has off farm income, if the household head is female, the travel time to nearest city, the dependency ratio, the hectares of agricultural land and ponds used by the households, and fixed effects at the upazila level. * $p<0.10$, ** $p<0.05$, *** $p<.01$.

Table 4: Regression analysis: correlates of economic and nutritional productivity, other stocked fish disaggregated.

**Unstocked fish** **Amount produced (t/ha)**	**Gross margin** (USD/ha) (1)	**kJ** (Annual AEs/ha) (2)	**Protein** (Annual AEs/ha) (3)	**Calcium** (Annual AEs/ha) (4)	**Iron** (Annual AEs/ha) (5)	**Zinc** (Annual AEs/ha) (6)	**Vitamin A** (Annual AEs/ha) (7)	**Vitamin B12** (Annual AEs/ha) (8)
**Clown knifefish**	81.9	9.4	6.8	-16.2	-10.1	0	-8.8	-94.5
	(8290.5)	(19.1)	(6.3)	(37.2)	(14.9)	(7.8)	(10.3)	(161.5)
**Snakehead**	1216.1	1.6	7.5***	5.9	5.5**	2.9**	-0.8	-38.9
	(1539.9)	(3.5)	(1.2)	(6.9)	(2.8)	(1.5)	(1.9)	(30.0)
**Tengra**	4276.2**	0	9.7***	47.7***	16.9***	9.1***	-2.1	260.6***
	(1931.3)	(4.4)	(1.5)	(8.7)	(3.5)	(1.8)	(2.4)	(37.6)
**Barramundi**	744.1	4.5**	7.8***	19.4***	7.1***	-0.4	0.3	44.6***
	(851.3)	(2.0)	(0.6)	(3.8)	(1.5)	(0.8)	(1.1)	(16.6)
**Bata**	1408	3.9	8.4***	9.4	2.8	3.4**	-3.4*	-73.7***
	(1453.5)	(3.3)	(1.1)	(6.5)	(2.6)	(1.4)	(1.8)	(28.3)
**Mola**	1969.6	2.9	11.4***	39.2***	19.6***	9.2***	66.9***	57.6
	(2144.8)	(4.9)	(1.6)	(9.6)	(3.9)	(2.0)	(2.7)	(41.8)
**Pool barb**	3544.6**	5.4	3.9***	-2.9	-7.3***	3.6**	2.8	52.8*
	(1520.1)	(3.5)	(1.2)	(6.8)	(2.7)	(1.4)	(1.9)	(29.6)
**Grey mullet**	-4580.3***	9.2***	7.9***	6.3	4	2	-0.1	-68.1**
	(1440.4)	(3.3)	(1.1)	(6.5)	(2.6)	(1.4)	(1.8)	(28.1)
**Bhangan**	-6933.0**	7.7	4.4*	-21.2	-6.1	11.0***	-0.7	-411.9***
	(3497.0)	(8.0)	(2.7)	(15.7)	(6.3)	(3.3)	(4.4)	(68.1)
**Yellowfin seabream**	15532.5	-0.1	6.5	-1.2	-1.4	4.3	-0.4	-113
	(11268.7)	(25.9)	(8.6)	(50.5)	(20.3)	(10.6)	(14.0)	(219.5)
**Spotted scat**	3301.9	5.6	6.4	-16.8	-2.6	0.8	-1.5	2
	(6881.2)	(15.8)	(5.2)	(30.8)	(12.4)	(6.5)	(8.6)	(134.0)
**Corsula**	-10616.7***	-17.7***	17.5***	-64.0***	-13.2***	0.6	-7.1**	6.5
	(2635.5)	(6.1)	(2.0)	(11.8)	(4.7)	(2.5)	(3.3)	(51.3)
**Others**	1283	13.2	3.7	-7.4	-1.3	-0.2	-0.3	24.7
	(3702.8)	(8.5)	(2.8)	(16.6)	(6.7)	(3.5)	(4.6)	(72.1)
**Observations**	**700**	**700**	**700**	**700**	**700**	**700**	**700**	**700**

Note: The dependent variable is the number of annual AEs of each nutrient produced per hectare. All models are OLS regressions that control for production amounts of other food groups: carp, other stocked fish, crustaceans, rice, leafy vegetables, vitamin A vegetables, other vegetables, root vegetables, vitamin A fruit, other fruit, nuts and oilseeds, as well as household head education, number of household members, household head age, a binary indicator if the household has off farm income, if the household head is female, the travel time to nearest city, the dependency ratio, the hectares of agricultural land and ponds used by the households, and fixed effects at the upazila level. * p<0.10, ** p<0.05, *** p<.01.

Table 5: Regression analysis: correlates of economic and nutritional productivity, unstocked fish disaggregated.

Crustaceans<br><br><br>Amount produced (t/ha)	Gross margin (USD/ha)<br>(1)	kJ (Annual AEs/ha)<br>(2)	Protein (Annual AEs/ha)<br>(3)	Calcium (Annual AEs/ha)<br>(4)	Iron (Annual AEs/ha)<br>(5)	Zinc (Annual AEs/ha)<br>(6)	Vitamin A (Annual AEs/ha)<br>(7)	Vitamin B12 (Annual AEs/ha)<br>(8)
**Black tiger shrimp**	2943.8***	0.4	4.8***	-1.9	3.3***	2.7***	-0.6	20.1***
	(332.1)	(0.8)	(0.3)	(1.6)	(0.6)	(0.3)	(0.6)	(6.9)
**Giant freshwater prawn**	7892.4***	0.6	4.9***	0.9	-1.4	1.1**	1.8*	73.9***
	(514.6)	(1.2)	(0.4)	(2.5)	(1.0)	(0.5)	(1.0)	(10.7)
**Indian white prawn**	-17227.1	23.5	9.9	-21.3	53.3	-3.5	7.8	131.2
	(18021.6)	(42.9)	(14.3)	(86.1)	(34.0)	(18.1)	(34.1)	(373.4)
**Spotted shrimp**	2516.4***	1.1	5.9***	12.1***	3.8***	3.6***	-1.8	16.5
	(667.3)	(1.6)	(0.5)	(3.2)	(1.3)	(0.7)	(1.3)	(13.8)
**Mud crab**	448.3	-2.6	2.0*	-3.3	-1.2	2.4*	-1.7	0.5
	(1305.9)	(3.1)	(1.0)	(6.2)	(2.5)	(1.3)	(2.5)	(27.1)
**Observations**	**700**	**700**	**700**	**700**	**700**	**700**	**700**	**700**

Note: The dependent variable is the number of annual AEs of each nutrient produced per hectare. All models are OLS regressions that control for production amounts of other food groups: carp, other stocked fish, unstocked fish, rice, leafy vegetables, vitamin A vegetables, other vegetables, root vegetables, vitamin A fruit, other fruit, nuts and oilseeds, as well as household head education, number of household members, household head age, a binary indicator if the household has off farm income, if the household head is female, the travel time to nearest city, the dependency ratio, the hectares of agricultural land and ponds used by the households, and fixed effects at the upazila level. * p<0.10, ** p<0.05, *** p<.01.

Table 6: Regression analysis: correlates of economic and nutritional productivity, crustaceans disaggregated.

**Other vegetables**<br>**Amount produced (t/ha)**	**Gross margin** (USD/ha) (1)	**kJ** (Annual AEs/ha) (2)	**Protein** (Annual AEs/ha) (3)	**Calcium** (Annual AEs/ha) (4)	**Iron** (Annual AEs/ha) (5)	**Zinc** (Annual AEs/ha) (6)	**Vitamin A** (Annual AEs/ha) (7)	**Vitamin B12** (Annual AEs/ha) (8)
**Chili**	-905.4	3.8	-1	-12.3	0	2.3	-0.1	-13.6
	(2398.4)	(5.4)	(1.6)	(10.8)	(3.4)	(2.2)	(4.3)	(47.7)
**Egg plant**	224	0.6	0.8***	1.3	0.8*	1.4***	0.5	-1.4
	(343.7)	(0.8)	(0.2)	(1.6)	(0.5)	(0.3)	(0.6)	(6.8)
**Okra**	-845.8	1.9	1.9***	0.9	3.5**	2.2**	2.9	5.4
	(1041.6)	(2.3)	(0.7)	(4.7)	(1.5)	(1.0)	(1.8)	(20.7)
**Ridge gourd**	167.5	0.1	0.5**	0.4	2.3***	1.1***	-0.1	-0.1
	(331.4)	(0.7)	(0.2)	(1.5)	(0.5)	(0.3)	(0.6)	(6.6)
**Bitter gourd**	126.6	0.4*	1.0***	0.1	3.4***	0.8***	0.5***	-0.3
	(97.9)	(0.2)	(0.1)	(0.4)	(0.1)	(0.1)	(0.2)	(1.9)
**Snake gourd**	456.8	0.3	0.4	0.5	2.9***	1.0***	0.4	1.9
	(391.9)	(0.9)	(0.3)	(1.8)	(0.6)	(0.4)	(0.7)	(7.8)
**Bottle gourd**	66.4	0.1	0.6***	1.4***	1.4***	1.4***	-0.2	2
	(112.4)	(0.3)	(0.1)	(0.5)	(0.2)	(0.1)	(0.2)	(2.2)
**Tomato**	92.4	0.2*	0.5***	0.3	0.3***	1.1***	0.3***	0.1
	(60.5)	(0.1)	(0.0)	(0.3)	(0.1)	(0.1)	(0.1)	(1.2)
**Cucumber**	256.3**	0.2	0.3***	-0.3	0.9***	0.4***	0.3	-0.9
	(114.5)	(0.3)	(0.1)	(0.5)	(0.2)	(0.1)	(0.2)	(2.3)
**Radish**	-626.7	-3.1	1.6	-0.3	-0.4	1.8	-2.4	27
	(2312.7)	(5.2)	(1.6)	(10.4)	(3.3)	(2.1)	(4.1)	(45.9)
**Long bean**	122.8	0.3	2.1***	2	3.0***	1.4***	0.2	1.7
	(313.0)	(0.7)	(0.2)	(1.4)	(0.4)	(0.3)	(0.6)	(6.2)
**Cabbage**	278.4	0.1	0.9**	1.7	1.3	1.1*	0.4	0.3
	(678.1)	(1.5)	(0.5)	(3.1)	(1.0)	(0.6)	(1.2)	(13.5)
**Cauliflower**	1643.8	-2.1	0.6	8.1	3	0.1	0.2	0.8
	(2522.2)	(5.7)	(1.7)	(11.4)	(3.6)	(2.3)	(4.5)	(50.1)
**Moringa**	-186.6	3	3.4	-6.1	0.6	-0.1	2.8	38.7
	(5687.1)	(12.8)	(3.9)	(25.7)	(8.1)	(5.2)	(10.1)	(113.0)
**Cowpea**	0	0	0	0	0	0	0	0
	(.)	(.)	(.)	(.)	(.)	(.)	(.)	(.)
**Other**	-847.6	1	1.2	-0.1	1.4	3.1	2.2	1.8
	(3653.9)	(8.2)	(2.5)	(16.5)	(5.2)	(3.4)	(6.5)	(72.6)
**Observations**	**700**	**700**	**700**	**700**	**700**	**700**	**700**	**700**

Note: The dependent variable is the number of annual AEs of each nutrient produced per hectare. All models are OLS regressions that control for production amounts of other food groups: carp, other stocked fish, unstocked fish, crustaceans, rice, leafy vegetables, vitamin A vegetables, root vegetables, vitamin A fruit, other fruit, nuts and oilseeds, as well as household head education, number of household members, household head age, a binary indicator if the household has off farm income, if the household head is female, the travel time to nearest city, the dependency ratio, the hectares of agricultural land and ponds used by the households, and fixed effects at the upazila level. * p<0.10, ** p<0.05, *** p<.01.

Table 7: Regression analysis: correlates of economic and nutritional productivity, other vegetables disaggregated.

Vitamin A fruit	Gross margin (USD/ha)	kJ (Annual AE/ha)	Protein (Annual AE/ha)	Calcium (Annual AE/ha)	Iron (Annual AE/ha)	Zinc (Annual AE/ha)	Vitamin A (Annual AE/ha)	Vitamin B12 (Annual AE/ha)
Amount produced (t/ha)	(1)	(2)	(3)	(4)	(5)	(6)	(7)	(8)
**Papaya**	-742.0**	3.1***	-0.1	-0.6	0.2	-0.1	-0.8	-6.9
	(363.1)	(0.8)	(0.3)	(1.7)	(0.7)	(0.3)	(0.6)	(7.2)
**Mango**	413.3	-0.5	-0.5	2.4	0	0.7	5.7***	4.2
	(528.6)	(1.2)	(0.4)	(2.4)	(1.0)	(0.5)	(0.9)	(10.5)
**Observations**	**700**	**700**	**700**	**700**	**700**	**700**	**700**	**700**

Note: The dependent variable is the number of annual AEs of each nutrient produced per hectare. All models are OLS regressions that control for production amounts of other food groups: carp, other stocked fish, unstocked fish, crustaceans, rice, leafy vegetables, vitamin A vegetables, other vegetables, root vegetables, other fruit, nuts and oilseeds, as well as household head education, number of household members, household head age, a binary indicator if the household has off farm income, if the household head is female, the travel time to nearest city, the dependency ratio, the hectares of agricultural land and ponds used by the households, and fixed effects at the upazila level. * $p<0.10$, ** $p<0.05$, *** $p<.01$.

Table 8: Regression analysis: correlates of economic and nutritional productivity, vitamin A fruit disaggregated.

Other fruit<br>Amount produced (t/ha)	Gross margin (USD/ha)<br>(1)	kJ (Annual AEs/ha)<br>(2)	Protein (Annual AEs/ha)<br>(3)	Calcium (Annual AEs/ha)<br>(4)	Iron (Annual AEs/ha)<br>(5)	Zinc (Annual AEs/ha)<br>(6)	Vitamin A (Annual AEs/ha)<br>(7)	Vitamin B12 (Annual AEs/ha)<br>(8)
**Banana**	-53.7	1.3***	0.5***	-0.4	0.7***	0.3***	-0.1	-1.8
	(127.0)	(0.3)	(0.1)	(0.6)	(0.2)	(0.1)	(0.2)	(2.5)
**Jackfruit**	481.9	0.1	0.2	0	0.4	0.6**	1.4***	5.9
	(292.7)	(0.7)	(0.2)	(1.3)	(0.5)	(0.3)	(0.5)	(5.8)
**Guava**	2292.4	0	-2.5	-26	-12.2	-2.3	3.2	-2.2
	(5802.3)	(13.1)	(4.4)	(26.5)	(10.3)	(5.6)	(10.4)	(115.5)
**Watermelon**	-3880.9	-14.6	7.1	-3.7	0.8	6.7	-0.2	126.6
	(32062.4)	(72.1)	(24.3)	(146.4)	(57.2)	(30.8)	(57.4)	(638.4)
**Hog plum**	803.3**	-0.1	0.6**	1.4	4.0***	0.5	0.5	5.1
	(358.9)	(0.8)	(0.3)	(1.6)	(0.6)	(0.3)	(0.6)	(7.1)
**Observations**	**700**	**700**	**700**	**700**	**700**	**700**	**700**	**700**

Note: The dependent variable is the number of annual AEs of each nutrient produced per hectare. All models are OLS regressions that control for production amounts of other food groups: carp, other stocked fish, unstocked fish, crustaceans, rice, leafy vegetables, vitamin A vegetables, other vegetables, root vegetables, vitamin A fruit, nuts and oilseeds, as well as household head education, number of household members, household head age, a binary indicator if the household has off farm income, if the household head is female, the travel time to nearest city, the dependency ratio, the hectares of agricultural land and ponds used by the households, and fixed effects at the upazila level. * p<0.10, ** p<0.05, *** p<.01.

Table 9: Regression analysis: correlates of economic and nutritional productivity, other fruit disaggregated.

**Nuts and oilseeds** **Amount produced (t/ha)**	**Gross margin** (USD/ha) (1)	**kJ** (Annual AEs/ha) (2)	**Protein** (Annual AEs/ha) (3)	**Calcium** (Annual AEs/ha) (4)	**Iron** (Annual AEs/ha) (5)	**Zinc** (Annual AEs/ha) (6)	**Vitamin A** (Annual AEs/ha) (7)	**Vitamin B12** (Annual AEs/ha) (8)
**Betel nut**	387	4.4***	2.6***	10.2***	10.3***	0.1	2.2***	2.6
	(286.1)	(0.6)	(0.2)	(1.3)	(0.5)	(0.2)	(0.5)	(5.7)
**Coconut**	349.7*	3.5***	1.3***	2.7***	4.1***	1.9***	1.1***	2.6
	(211.5)	(0.5)	(0.1)	(1.0)	(0.3)	(0.1)	(0.4)	(4.2)
**Mustard seed**	2794.4***	6.5***	11.2***	6.1	19.0***	15.7***	0.9	2.8
	(900.0)	(2.0)	(0.6)	(4.0)	(1.5)	(0.6)	(1.6)	(17.9)
**Observations**	**700**	**700**	**700**	**700**	**700**	**700**	**700**	**700**

Note: The dependent variable is the number of annual AEs of each nutrient produced per hectare. All models OLS regressions that control for household head education, number of household members, household head age, a binary indicator if the household has off farm income, if the household head is female, the travel time to nearest city, the dependency ratio, the hectares of agricultural land and ponds used by the households, and fixed effects at the upazila level. * p<0.10, ** p<0.05, *** p<.01.

Table 10: Regression analysis: correlates of economic and nutritional productivity, nuts and oilseeds disaggregated

SI References
