# Supplementary material for: Integrated aquatic and terrestrial food production enhances micronutrient and economic productivity for nutrition-sensitive food systems
Source: Nat Food. 2023 Sep 4;4(10):866–73. doi: 10.1038/s43016-023-00840-8 (PMC10589083; doi:10.1038/s43016-023-00840-8)
Supplement: Supplementary file 2 — Reporting Summary [file 43016_2023_840_MOESM2_ESM.pdf]

nature portfolio

Corresponding author(s): Liz Ignowski

Last updated by author(s): Aug 2, 2023

Reporting Summary

Nature Portfolio wishes to improve the reproducibility of the work that we publish. This form provides structure for consistency and transparency in reporting. For further information on Nature Portfolio policies, see our Editorial Policies and the Editorial Policy Checklist.

Statistics

For all statistical analyses, confirm that the following items are present in the figure legend, table legend, main text, or Methods section.

n/a	Confirmed	
☐	☒	The exact sample size (*n*) for each experimental group/condition, given as a discrete number and unit of measurement
☐	☒	A statement on whether measurements were taken from distinct samples or whether the same sample was measured repeatedly
☒	☐	The statistical test(s) used AND whether they are one- or two-sided<br>*Only common tests should be described solely by name; describe more complex techniques in the Methods section.*
☐	☒	A description of all covariates tested
☒	☐	A description of any assumptions or corrections, such as tests of normality and adjustment for multiple comparisons
☒	☐	A full description of the statistical parameters including central tendency (e.g. means) or other basic estimates (e.g. regression coefficient) AND variation (e.g. standard deviation) or associated estimates of uncertainty (e.g. confidence intervals)
☐	☒	For null hypothesis testing, the test statistic (e.g. *F*, *t*, *r*) with confidence intervals, effect sizes, degrees of freedom and *P* value noted<br>*Give P values as exact values whenever suitable.*
☒	☐	For Bayesian analysis, information on the choice of priors and Markov chain Monte Carlo settings
☒	☐	For hierarchical and complex designs, identification of the appropriate level for tests and full reporting of outcomes
☒	☐	Estimates of effect sizes (e.g. Cohen's *d*, Pearson's *r*), indicating how they were calculated

Our web collection on statistics for biologists contains articles on many of the points above.

Software and code

Policy information about availability of computer code

Data collection	The household survey data was collected using KoBoToolbox, v1.28, a free open source platform, by our research team and enumerators in Bangladesh.
Data analysis	Descriptive statistics and regression analyses were computed in StataSE Version 17.

For manuscripts utilizing custom algorithms or software that are central to the research but not yet described in published literature, software must be made available to editors and reviewers. We strongly encourage code deposition in a community repository (e.g. GitHub). See the Nature Portfolio guidelines for submitting code & software for further information.

Data

Policy information about availability of data

All manuscripts must include a data availability statement. This statement should provide the following information, where applicable:

- Accession codes, unique identifiers, or web links for publicly available datasets
- A description of any restrictions on data availability
- For clinical datasets or third party data, please ensure that the statement adheres to our policy

The two nutrient datasets of the Bangladesh Food Composition Table and nutrient composition of fish species are publicly available at https://www.fao.org/fileadmin/templates/food_composition/documents/FCT_10_2_14_final_version.pdf and https://doi.org/10.1371/journal.pone.0175098. Survey data on [WorldFish

data, please specify details] will be publicly available via https://doi.org/10.7910/DVN/5HLK4G as of 26 March 2024 and until then it will be shared upon request to the corresponding author.

Human research participants

Policy information about studies involving human research participants and Sex and Gender in Research.

Reporting on sex and gender	The survey was a household level survey focused on aquaculture. We collected data on the household roster but the household head/person with the most knowledge reported on the aquaculture farming activities.
Population characteristics	See "Behavioural & social sciences study design" below.
Recruitment	Survey data was collected in December 2020 and January 2021 using KoBoToolbox for the second round of a panel survey first conducted in 2013. In each of seven selected districts, all sub-districts (upazila) with non-negligible aquaculture production were included in the initial sample frame of the original survey, then selected randomly by proportional probability sampling (PPS). In each selected upazila, all mouza (the smallest administrative unit reported in the Bangladesh agricultural census), underwent a second stage of trimming to eliminate those with fewer than 20 aquaculture farms, as reported in the national agricultural census of 2008 (the most recently available agricultural census for Bangladesh). Two to three mouza were then selected randomly from each upazila. Prior to the survey, a census of fish farmers was conducted in all selected mouza, among which 20 farms were selected randomly for interview.
Ethics oversight	The Michigan State University Institutional Review Board determined this study (STUDY00003689) to be exempt under 45 CFR 46.104(d) 2(ii).

Note that full information on the approval of the study protocol must also be provided in the manuscript.

Field-specific reporting

Please select the one below that is the best fit for your research. If you are not sure, read the appropriate sections before making your selection.

☐ Life sciences ☒ Behavioural & social sciences ☐ Ecological, evolutionary & environmental sciences

For a reference copy of the document with all sections, see nature.com/documents/nr-reporting-summary-flat.pdf

Behavioural & social sciences study design

All studies must disclose on these points even when the disclosure is negative.

Study description	This is a quantitative case study on nutrient productivity by different aquatic farming systems in Bangladesh. This study calculates the economic and nutrient productivity per area for different types of integrated aquatic farming systems in order to demonstrate the importance of nutrition-sensitive agricultural approaches.
Research sample	Bangladeshi households within the study area that had a sample pond that had been used for aquaculture within the past 12 months prior to the survey. The respondents were a household member in the house at the time of interview who had detailed knowledge of aquaculture and household activities. Our sample was representative of households in Southwest Bangladesh.
Sampling strategy	This survey was the second round of a panel survey first conducted in 2013. In each of seven selected districts, all sub-districts (upazila) with non-negligible aquaculture production were included in the initial sample frame of the original survey, then selected randomly by proportional probability sampling (PPS). In each selected upazila, all mouza (the smallest administrative unit reported in the Bangladesh agricultural census), underwent a second stage of trimming to eliminate those with fewer than 20 aquaculture farms, as reported in the national agricultural census of 2008 (the most recently available agricultural census for Bangladesh). Two to three mouza were then selected randomly from each upazila. Prior to the survey, a census of fish farmers was conducted in all selected mouza, among which 20 farms were selected randomly for interview.<br><br>In 2020 we conducted a new farm census in each mouza included in the 2013 survey. All farms included in the previous survey round that could be contacted and gave their consent to be interviewed were resurveyed. The rate of attrition between the two survey rounds was approximately 20%. All missing farms were replaced at random with others selected from the updated census list. During the 2020 survey, detailed production data were collected from a single 'sample parcel' that had been used for aquaculture within the past 12 months, whether or not integrated with terrestrial foods. Where households operated more than one plot of aquaculture land, the sample parcel was selected at random from among these. The sample thus represents the entire population of aquaculture farms in the seven selected districts.
Data collection	The use of Kobo toolbox was designed to stream-line the data collection process from the field.<br>Enumerators linked to their supervisor account and completed interviews were synced at the end of each day through a Wi-Fi connection to a server. Because the data was available daily it was monitored closely throughout the entire data collection period.

	There may have been other household members present at the time of the interview. However, as there were no experimental conditions, we do not assume that this had any influence on our results.
Timing	Survey data was collected in December 2020 and January 2021.
Data exclusions	No data were excluded from analysis.
Non-participation	Very few households refused to participate, less that 20 instances with the main reason being time constraints.
Randomization	Participants were not allocated into experimental groups.

Reporting for specific materials, systems and methods

We require information from authors about some types of materials, experimental systems and methods used in many studies. Here, indicate whether each material, system or method listed is relevant to your study. If you are not sure if a list item applies to your research, read the appropriate section before selecting a response.

Materials & experimental systems

n/a	Involved in the study
☒	☐ Antibodies
☒	☐ Eukaryotic cell lines
☒	☐ Palaeontology and archaeology
☒	☐ Animals and other organisms
☒	☐ Clinical data
☒	☐ Dual use research of concern

Methods

n/a	Involved in the study
☒	☐ ChIP-seq
☒	☐ Flow cytometry
☒	☐ MRI-based neuroimaging
